# Tranexamic acid in the management of postpartum hemorrhage following vacuum-assisted vaginal delivery in primiparous women: a retrospective cohort study

**DOI:** 10.1186/s12884-025-08438-7

**Published:** 2025-12-29

**Authors:** Orna Reichman, Amal Yousef, Tal Margaliot, Maayan Bas Lando, Sarit Helman, Vladimir Plotkin, Sorina Grisaru-Granovsky

**Affiliations:** Department of Obstetrics and Gynaecology, The Eisenberg R&D Authority, Shaare Zedek Medical Centre, Faculty of Medicine, Hebrew University of Jerusalem, Jerusalem, Israel

**Keywords:** Postpartum hemorrhage, Vacuum-assisted vaginal delivery, Tranexamic acid, Primiparas

## Abstract

**Background:**

To evaluate whether tranexamic acid (TXA) administration reduces the prevalence of severe postpartum hemorrhage (sPPH), defined as a hemoglobin drop of ≥ 3 g/dL, in primiparous women undergoing vacuum-assisted vaginal delivery (VAVD).

**Methods:**

A retrospective cohort study was conducted at a large tertiary medical center, including all primiparous women undergoing VAVD between January 2021 and December 2022. TXA (1 g IV within 30 min of delivery) was administered at the discretion of the attending clinician, such that some women received TXA while others did not. The primary outcome was sPPH. Secondary outcomes included postpartum transfusion of blood products, absolute decline in hemoglobin levels, and additional clinical interventions related to hemorrhage, such as manual removal of the placenta or administration of uterotonic agents for the treatment of uterine atony. Initial comparisons were performed between TXA-treated and untreated women in the overall cohort. To account for baseline differences in the likelihood of receiving TXA, propensity score matching was performed using relevant clinical predictors; neonatal birthweight, prolonged second or third stage of labor, manual uterine revision. Logistic regression models were used for adjusted analyses.

**Results:**

During the study period, 6,580 primiparous women delivered, of whom 1,048 (15.9%) met the inclusion criteria and comprised the study cohort (*N* = 1,048). Of these, 383 (36.5%) received TXA, and 274 (26.1%) experienced sPPH. TXA-treated women had higher sPPH rates compared to untreated women (33.5% vs. 22.1%, *p* < 0.001), greater mean hemoglobin drop (2.54 ± 1.3 vs. 2.18 ± 1.3 g/dL, *p* < 0.001), and increased postpartum blood transfusion rates (3.7% vs. 1.5%, *p* = 0.031). Propensity score matching (367 pairs) yielded similar results, with sPPH remaining more prevalent in the TXA group (31.7% vs. 18.8%, *p* < 0.001).

**Conclusions:**

Primiparous women undergoing VAVD are at increased risk for sPPH. Administration of 1 gram of TXA within 30 min of delivery was not associated with a reduction in the prevalence of sPPH or the need for postpartum blood transfusion. Given the non-randomized design and retrospective nature of the study, it was not possible to determine whether TXA was administered prophylactically or in response to active bleeding. Nevertheless, TXA did not appear to reduce the prevalence of sPPH. Further research is needed to identify effective interventions for sPPH prevention in this high-risk population.

## Background

Postpartum hemorrhage (PPH) is a leading cause of maternal mortality globally, especially in low-resource settings [1, 2]. In 2020, an estimated 27% of the 250,000 maternal deaths reported in sub-Saharan Africa and South Asia were attributable to PPH [3]. Although maternal mortality from PPH is comparatively rare in high-income countries, the condition is nonetheless associated with considerable maternal morbidity, including severe anemia, increased requirements for blood transfusion, surgical interventions, intensive care unit admissions, and prolonged hospitalization [4].

Tranexamic acid (TXA) is a widely used antifibrinolytic agent, administered both prophylactically and therapeutically to manage hemorrhage across a broad spectrum of surgical and trauma settings. [5–8] Robust evidence supports its efficacy in reducing blood loss and mortality in surgical procedures including cesarean delivery (CD), [6] as well as in the management of hemorrhage following trauma. [7, 8] In obstetrics, however, large randomized controlled trials have not demonstrated a benefit for the routine prophylactic use of TXA[9–11].

The efficacy of TXA in vaginal delivery remains a subject of ongoing investigation and debate, as existing studies vary widely in application (prophylactic versus therapeutic) and in the baseline PPH risk of enrolled populations[9–15]. Most randomized controlled trials in unselected women undergoing vaginal delivery have consistently shown no effect of prophylactic TXA, and meta-analyses likewise report no significant reduction in severe PPH[9–15]. Large randomized trials in high-risk populations for PPH, such as the WOMAN-2 trial in women with anemia, have similarly shown that prophylactic TXA does not reduce the incidence of PPH[16]. Conversely, a recent RCT conducted in women with mixed PPH risk levels demonstrated a significant reduction in both sPPH and postpartum blood transfusion rates following TXA administration[17]. To date, however, no large observational study has evaluated the benefit of TXA administration in a selective high-risk subgroup, such as primiparous women undergoing vacuum-assisted vaginal delivery (VAVD), with reported sPPH rates approaching 27%[18].Accordingly, the objective of this retrospective cohort was to evaluate whether TXA administration was associated with the prevalence of sPPH, defined as a hemoglobin drop of ≥ 3 g/dL among primiparous women undergoing VAVD.

## Materials and methods

### Study Design

A retrospective cohort, including primiparous women with singleton pregnancies who underwent VAVD at Shaare Zedek Medical Center (SZMC) between January 2021 and December 2022. The study was approved by the SZMC Institutional Review Board (IRB #0329 − 24).

### Setting

SZMC is a tertiary university-affiliated hospital and the largest maternity center in Israel, with approximately 19,000 deliveries performed annually. The parity distribution at SZMC comprises 20% primiparous women and 18% grand multiparous women (parity ≥ 6). CDs constitute 12% of deliveries, while VAVD comprise 5%; forceps are not performed in our department. Notably, over 95% of the deliveries are publicly funded for the mother and neonate by the National Health Insurance system. Obstetric care is predominantly delivered by midwives and resident physicians, under the direct supervision of board-certified obstetricians.

At our institution, VAVD are conducted by either a senior obstetrician or a senior resident with a minimum of three years of training in line with national guidelines that restrict the procedure to cases with a fetal head at station + 2 or lower and a gestational age of ≥ 34 weeks. The choice of vacuum device; Kiwi cup, soft cup, or metal vacuum cap, is guided by clinical judgment. In accordance with institutional protocol, a mediolateral episiotomy is routinely performed in all primiparous women undergoing vacuum delivery and they all prophylactically receive 10 IU of intramuscular oxytocin immediately after delivery.

### Eligibility criteria

Included were all primiparous women who underwent VAVD during the study period. Women with multiple gestations, failed vacuum deliveries, or missing records of pre- or post-delivery hemoglobin values were excluded.

### Exposure classification

Throughout the study period, the administration of intravenous TXA, 1 gram for the prevention or management of PPH, was not standardized and was left to the discretion of the attending obstetrician. When administered, TXA was given within 30 min of delivery. Owing to the retrospective design, it was not possible to reliably distinguish between prophylactic use and treatment initiated in response to acute PPH. We considered it inappropriate to assume that all women ultimately diagnosed with PPH had received TXA, since real-time visual estimation of blood loss tends to underestimate actual volume and has limited sensitivity for identifying PPH[19].

### Outcome measures

The primary outcome was sPPH, defined as a hemoglobin decline of ≥ 3 g/dL during the index hospitalization. This definition was selected because hemoglobin change provides an objective and quantifiable surrogate for blood loss, particularly in retrospective studies where direct measurement is often inaccurate or incomplete. Evidence indicates that a hemoglobin decrease of ≥ 2 g/dL is frequently observed in women with blood loss ≥ 1,000 mL [20]; therefore, although not a formal diagnostic criterion, a decline of ≥ 3 g/dL can reasonably be inferred to correspond to blood loss exceeding 1,000 mL. Hemoglobin drop was calculated as the difference between the maximum and minimum hemoglobin values recorded from admission to the delivery ward and through discharge. Secondary outcomes included blood product transfusion, absolute delta hemoglobin (g/dL), and any additional clinical interventions related to hemorrhage management, such as manual uterine revision or placental removal, additional administration of uterotonics, or bleeding control surgical procedures.

### Data collection

 Data was extracted from a hospital computerized database that is updated in real time by attending midwives and obstetricians during labor and delivery. Most variables in the electronic medical record are entered into fixed, mandatory fields, ensuring data completeness prior to patient transfer to the postpartum ward. Variables included maternal demographics, labor characteristics, interventions, neonatal outcomes, TXA administration, and hemoglobin levels. A manual chart review was conducted to confirm that TXA was administered and to document the clinical impression of bleeding severity as noted by the attending midwife and obstetrician. When applicable, documentation was also reviewed for evidence suggestive of uterine atony, as these variables were not routinely recorded in the structured fields.

### Statistical analysis

Baseline characteristics were compared using chi-square/Fisher’s exact test (categorical variables) or Student’s t-test (continuous variables). To address potential selection bias in TXA administration, a propensity score for TXA receipt was calculated based on known PPH risk factors including, manual uterine revision or placental removal, prolonged second or third stages of labor and neonatal birthweight as predictor variables. Each woman who received TXA was matched to a single woman who did not receive TXA using the closest available propensity score. Standardized mean differences (SMD) were assessed post-matching for balance. Logistic regression models were used to assess the association between TXA administration and primary outcome. Analyses were conducted using SPSS v30. *P* < 0.05 was considered statistically significant.

### Ethical considerations

 Given the retrospective nature, informed consent was waived. The study adheres to the STROBE guidelines [21].

## Results

**Fig. 1:**
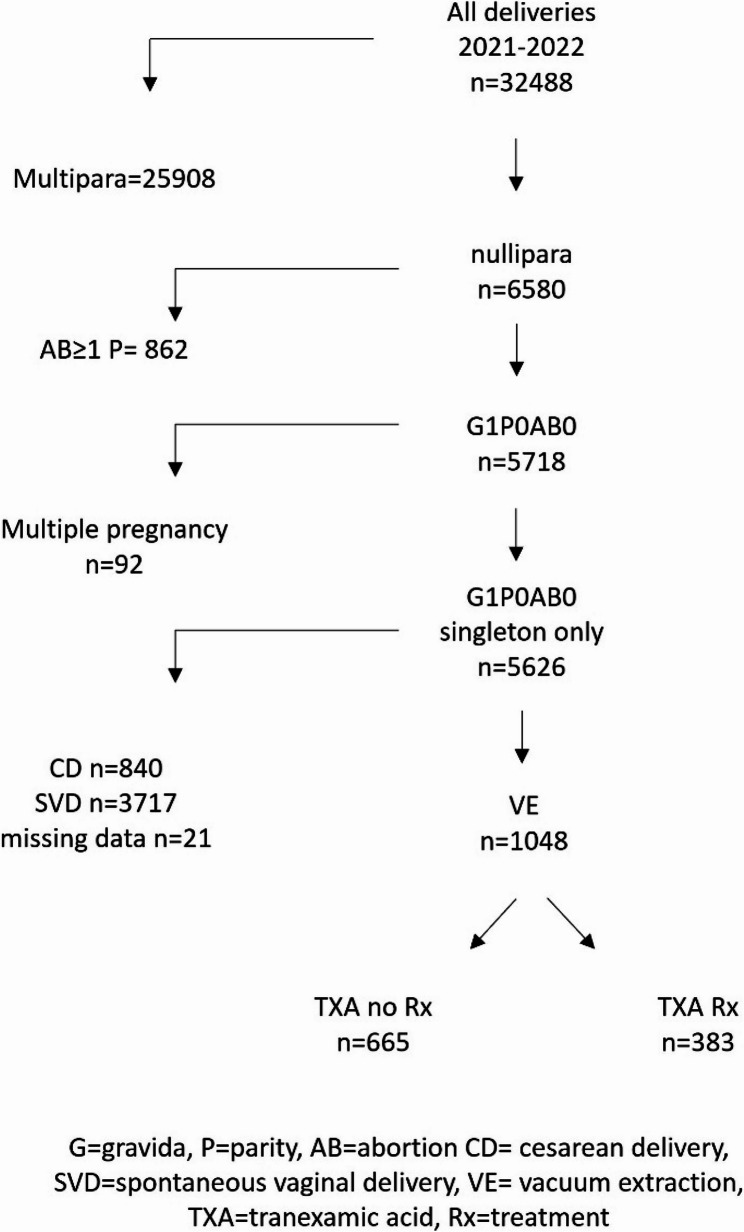
Flowchart of the study population

During the study period, 32,488 deliveries were recorded, of which 6,580 (20.3%) were among primiparous women. After excluding women with a prior miscarriage or abortion, multiple gestations, cesarean delivery, or spontaneous onset of labor, 1,048 primiparous women (15.9%) who underwent VAVD were included and comprised the study cohort (*N* = 1,048). Among those, approximately one-third, 383 women (36.5%) received TXA treatment. A flowchart illustrating the study population is provided in Fig. 1.

No significant differences were found between the TXA-treated group and the untreated group regarding established risk factors for sPPH, such as mode of labor onset, duration of the third stage of labor, need for a manual uterine revision or placental removal, uterine atony, or neonatal birthweight. However, the second stage of labor was significantly longer in the TXA group (136 ± 76 min) compared to the non-TXA group (123 ± 73 min; *p* = 0.005). A prolonged second stage was documented in 129 women (34%) who received TXA, versus 177 women (27%) who did not (*p* = 0.017), as detailed in Table 1.

**Table 1:** Baseline maternal and obstetric characteristics of primiparous women undergoing vacuum-assisted vaginal delivery by TXA administration status (*N* = 1,048)

Variable	No TXA (*N* = 665)	TXA (*N* = 383)	*P* value
Maternal age (years, mean ± SD)	24.3 ± 3.9	24.7 ± 4.1	0.115
Education (> 12 years) n (%)	259 (56%)	141 (57%)	0.751
Gestational age (weeks ± SD)	39.6 ± 1.3	39.6 ± 1.2	0.361
Induction of labor n (%)	155 (23%)	83 (22%)	0.592
Epidural anesthesia n (%)	629 (96%)	358 (93%)	0.494
Neonatal sex (male) n (%)	377 (57%)	202 (53%)	0.221
Birthweight (g, mean ± SD)	3222 ± 404	3267 ± 405	0.086
Macrosomia (> 4000 g) n (%)	16 (2.4%)	16 (4.2%)	0.135
Second stage duration (min ± SD)	123 ± 73	136 ± 76	0.005
Prolonged 2nd stage n (%)†	177 (27%)	129 (34%)	0.017
Prolonged 3rd stage (≥ 20 min)	26 (4%)	20 (5%)	0.353
manual uterine revision or placental removal n (%)	15 (2.3%)	5 (1.3%)	0.352
Uterine atony n (%)	5 (0.8%)	8 (2.1%)	0.080
Apgar score at 5 min ≤ 7 n (%)	8 (1.2%)	4 (1.0%)	1.000

One in four primiparous women who underwent VAVD experienced sPPH, with a prevalence of 274 out of 1,048 cases (26.1%). Stratification by TXA treatment demonstrated that the primary outcome, sPPH, occurred significantly more often in the TXA-treated group compared with the untreated group (128 cases, 33.5% vs. 146 cases, 22.1%; *p* < 0.001). For the secondary outcomes, women who received TXA exhibited a larger mean hemoglobin decline (2.54 ± 1.3 vs. 2.18 ± 1.3 g/dL; *p* < 0.001) and a higher rate of postpartum blood transfusion (3.7% vs. 1.5%; *p* = 0.031), as shown in Table 2.

**Table 2:** Primary and secondary outcomes of primiparous women undergoing vacuum-assisted delivery, by TXA administration status

Outcome	No TXA (*N* = 665)	TXA (*N* = 383)	*P* value
Severe PPH (Hb drop ≥ 3 g/dL)	146 (22.1%)	128 (33.5%)	< 0.001
Blood transfusion	10 (1.5%)	14 (3.7%)	0.031
Mean delta hemoglobin (g/dL, mean ± SD)	2.18 ± 1.3	2.54 ± 1.3	< 0.001

### Propensity score matching

To reduce potential selection bias in TXA administration, a propensity score for receiving TXA was estimated using logistic regression. Predictor variables included established risk factors for PPH: neonatal birthweight, manual uterine revision or placental removal, and prolonged second or third stages of labor. Each woman who received TXA was matched to one woman who did not, based on the closest available propensity score within a caliper of 0.2 standard deviations. This procedure yielded 367 matched pairs (*N* = 734). The post-matching covariate balance was assessed via standardized mean differences (SMDs), with most variables showing a satisfactory balance (SMD < 0.1). However, manual uterine revision or placental removal remained an unbalanced factor, occurring more frequently in the TXA group (five cases vs. two in the untreated group; SMD = 0.3). In the matched analysis, sPPH prevalence remained significantly higher in the TXA-treated group (116 cases, 31.7%) compared to the untreated group (69 cases, 18.8%), *p* < 0.001.

## Discussion

The overall prevalence of sPPH in our cohort (26.1%) aligns with previous reports identifying primiparous women undergoing VAVD as a particularly high-risk group. We have previously introduced the P-C-MoD classification, in which 126,693 parturients were grouped into 12 subgroups according to Parity (primipara vs. multipara), prior **C**esarean delivery (previous CD versus unscarred uterus), and Mode of Delivery (spontaneous vaginal delivery vs. vacuum assisted vaginal delivery vs. CD), highlighting the increased risk of PPH in primiparas undergoing VAVD, with rates approaching 27%[18]. Similarly, Hiersch et al.[22] reported sPPH rates of 25.6% following VAVD in primiparous women. These consistent findings underscore the need for targeted preventive strategies in this subgroup. Several factors likely contribute to this elevated risk, including greater soft-tissue trauma, routine episiotomy, and prolonged second or third stages of labor, all of which predispose to bleeding. These features suggest a bleeding phenotype primarily driven by tissue injury and labor mechanics, in which TXA administered late or without clear targeting may be insufficient to offset ongoing hemorrhage. Practical aspects of workflow may also play a role: during busy shifts, limited staffing can result in delays between extraction and surgical repair of lacerations or episiotomies, further prolonging blood loss.

Although the crude prevalence of PPH in high-income countries is estimated at ~ 6%, the crude rate at our center is similar at 7%[23], emphasizing that even in a well-resourced tertiary university hospital, high-risk subgroups such as primiparous women undergoing VAVD remain disproportionately affected, with sPPH rates far exceeding the general background prevalence. Cross-study comparisons should be interpreted cautiously, given the heterogeneity in PPH definitions ranging from estimated blood loss to clinical or laboratory thresholds[24]. In this retrospective study, we relied on hemoglobin decline and transfusion requirements, which provide objective measures while minimizing subjectivity and reducing missing data. The role of TXA in the management of obstetric hemorrhage has been extensively investigated. However, the existing literature comprises heterogeneous study populations with varying baseline risk for sPPH. While some trials have evaluated the therapeutic use of TXA for established sPPH, others have assessed its prophylactic administration in general obstetric cohorts. This heterogeneity limits the ability to draw definitive conclusions regarding the overall effectiveness of TXA.

### Studies evaluating prophylactic TXA administration in low-risk populations for PPH

The landmark multicenter, double-blind RCT (the TRAAP trial), which randomized over 4,000 women with singleton vaginal deliveries, to receive prophylactic administration of TXA (1 g IV) immediately following delivery did not result in a statistically significant reduction in the incidence of severe PPH (≥ 1000 mL), with rates of 1.7% in the TXA group versus 2.1% in the placebo group (RR 0.81; 95% CI, 0.55–1.19)[9].

A recent Cochrane meta-analysis evaluated the efficacy of prophylactic TXA, administered in addition to standard care for the prevention of PPH. The analysis synthesized data from three RCTs, comprising 18,974 participants across diverse healthcare settings and encompassing women with varying baseline risks for PPH. Reported prevalence of PPH in the study’s population ranged between 7% and 10%. Prophylactic TXA was associated with a modest, non-significant reduction in the incidence of severe blood loss ≥ 1000 mL (RR 0.86, 95% CI 0.69–1.07; 2 trials, 18,897 participants). ¹³ These results align with a systematic review and IPD meta-analysis of five RCTs including 19,891 women, which concluded that TXA does not meaningfully reduce PPH or severe morbidity after vaginal delivery and does not increase thromboembolic risk[11]. According to the current evidence, TXA is not recommended as PPH prophylaxis during vaginal delivery.

### Studies evaluating prophylactic TXA administration in high-risk populations for PPH

In In the WOMAN-2 trial, 15,068 women with moderate or severe anemia who delivered vaginally were randomized to receive TXA or placebo within 15 min of cord clamping. The incidence of clinically diagnosed PPH was similar between groups (7.0% with TXA vs. 6.6% with placebo; RR 1.05, 95% CI 0.94–1.19). The study concluded that prophylactic TXA did not reduce the risk of PPH in this population[16]. By contrast, in the TRAAP trial, among 667 women who underwent operative vaginal delivery, treatment with TXA reduced the prevalence of PPH from 14.7% (48 cases) in the placebo group to 9.4% (32 cases), suggesting a potential benefit in high-risk populations[9]. Consistent findings were reported in a large RCTconducted in China that enrolled 2,258 women with one or more risk factors for PPH undergoing vaginal delivery. Participants were randomized to receive either 1 g intravenous TXA or placebo immediately after birth. Severe PPH, defined as blood loss ≥ 1000 mL within 24 h, occurred significantly less frequently in the TXA group (2.7%) compared to the placebo group (5.6%) (RR 0.49, 95% CI 0.32–0.74; *p*= 0.001)[17].

### TXA use for treating PPH

Given that our study examined both prophylactic and therapeutic uses of TXA, reference to the WOMAN trial is essential [25]. This large, international, double-blind, placebo-controlled RCT assessed the efficacy of TXA in women with clinically diagnosed sPPH, defined as bleeding with potential for hemodynamic compromise or requiring urgent intervention. The trial enrolled 14,219 women post-vaginal delivery and 5,836 post-CD. Among those delivering vaginally, no significant difference was observed in the composite primary endpoint (death or hysterectomy), 3.6% (255/7,080) in the TXA group versus 4.1% (288/7,108) in the placebo group (RR 0.89, 95% CI 0.75–1.05) [25].

The prevalence of sPPH in our cohort was notably high, reaching 26.1%, and appears to exceed the rate reported in women undergoing VAVD (*n* = 667) in the TRAAP trial, in which approximately 13% experienced PPH. Several factors may account for this discrepancy. First, the TRAAP study population comprised women of mixed parity. The distribution between primiparous and multiparous participants among those undergoing VAVD was not reported, limiting direct comparison. Second, the operational definition of PPH differs between the studies: the TRAAP trial defined PPH as ≥ 500 mL of blood loss measured immediately postpartum, whereas our study employed a more inclusive clinical definition of a hemoglobin drop of > 3 g/dL measured over the entire postpartum hospitalization. This methodological difference must be considered when comparing PPH prevalence across studies.

Another important distinction between our study and other previous randomized controlled trials concerns the timing and intent of TXA administration, prophylactic versus therapeutic. While landmark trials such as the TRAAP study and the recent Cochrane meta-analysis focused primarily on prophylactic administration of TXA to unselected populations immediately following vaginal delivery, our study reflects real-world clinical practice in which TXA was administered both prophylactically and therapeutically at the discretion of the attending provider in a high-risk population for PPH.

In conclusion, our findings confirm that primiparous women undergoing VAVD represent a high-risk population for postpartum hemorrhage. In this high-risk cohort of primiparous women undergoing VAVD, TXA administration, whether prophylactic or therapeutic, was not associated with lower rates of sPPH or postpartum transfusion; rather, higher rates were observed among treated women, likely reflecting selection bias and clinical practice patterns.

Further research is needed to identify more effective strategies for PPH prevention in primiparas undergoing VAVD.

#### Strengths and limitations

A major strength of this study lies in its focus on a clearly defined high-risk population, primiparous women undergoing VAVD, within a large, real-world cohort from a tertiary medical center with a high annual delivery volume. Comprehensive and standardized data collection through an electronic medical record system minimized missing data and enhanced data reliability. In addition, the use of propensity score matching allowed for adjustment of key confounders and improved the internal validity of the comparative analysis between TXA-treated and untreated groups.

However, several limitations must be acknowledged. First, the retrospective, observational design precludes causal inference and may be subject to residual confounding, despite the use of propensity score matching. Second, TXA administration was non-standardized and left to clinician discretion, introducing potential selection bias related to clinical judgment and intervention timing, sometimes allowing the TXA to be administrated at the beginning of a hemorrhage episode rather than prophylactic. This practice may have introduced selection bias, as women with more severe bleeding were more likely to receive TXA, which could in turn explain the higher rates of sPPH and transfusion observed in the TXA-treated group. Third, the definition of PPH was based on hemoglobin decline rather than direct quantification of blood loss, which may be influenced by peri-delivery fluid management and laboratory timing. Lastly, although this study reflects real-world clinical practice, its findings may not be generalizable to settings with different obstetric care protocols.

## Data Availability

The datasets generated and analyzed during the current study are not publicly available due to institutional policy, but are available from the corresponding author upon reasonable request.
